# Tanjun Tong: a pioneer of cellular senescence and aging mechanism research in China

**DOI:** 10.1093/procel/pwae024

**Published:** 2024-05-10

**Authors:** Cheng Zhen, Quanxiu Li

**Affiliations:** Center for the History of Medicine, School of Health Humanities, Peking University, Beijing 100191, China; School of Health Humanities, Peking University, Beijing 100191, China

Tanjun Tong (童坦君, 1934–2022) (Fig. 1), was a renowned biochemist in modern China, this year marks the 90th anniversary of the birth of him. He was elected as a member (academician) of the Chinese Academy of Sciences Division of Life Sciences and Medical Sciences in 2005. He completed his undergraduate and graduate studies in Beijing Medical College (now Peking University Health Science Center) and became a faculty member in Beijing Medical College. He was one of the 52 researchers to visit the United States from 1978 to 1981, the first group of visiting scholars to the USA. Since the founding of new China, he had worked on cancer biology and protein biochemistry, and then shifted his research focus to the elucidation of the molecular mechanisms of cellular senescence and aging, and established pioneering explorations in aging and gerontology research in China. He exhibited unmatched perseverance, great diligence, and high academic productivity all through his life, and published around 400 academic journal articles and multiple monographs. His dedication led to the founding of the Peking University Research Center on Aging, which made tremendous contributions to senescence and aging research in modern China.

**Figure 1.**
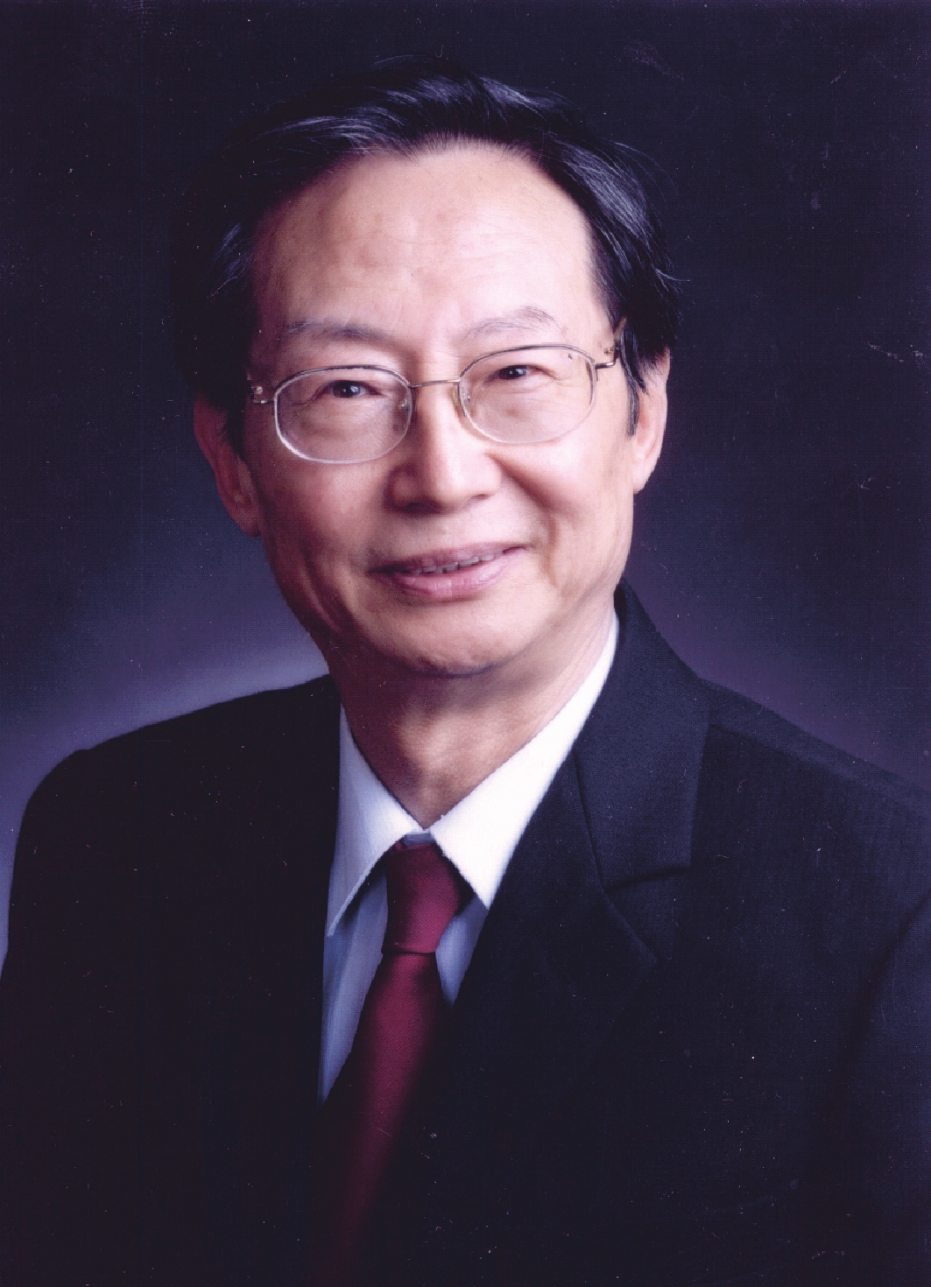
Professor Tanjun Tong (1934–2022).

## Early life and education

**Figure 2.**
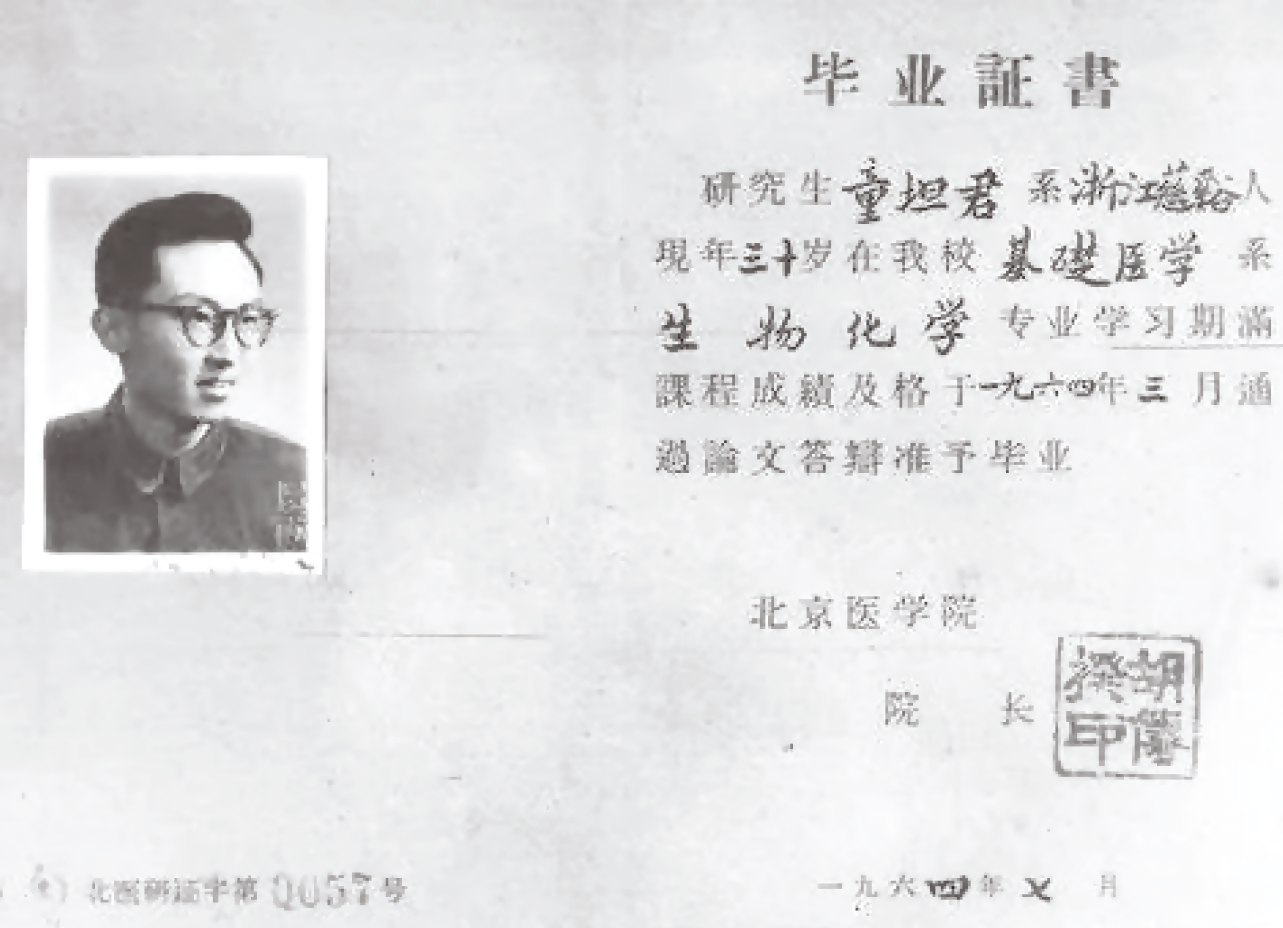
Graduation Certificate of Tanjun Tong from Beijing Medical College in 1964 (Courtesy of Peking University Health Science Center Archive).

Tanjun Tong was born in August 1934 in Cixi, Zhejiang Province, China. Tong’s ancestral family used to be prominent and wealthy, but no longer during his childhood. He suffered from starvation at times and developed the childhood disease rickets due to malnutrition at 4–5 years old. He moved to Shanghai to attend a Christian primary school in 1942, and was later admitted to Shanghai Yucai Junior High School (上海市立育才中学). He was infected with tuberculosis during his second year in junior high school in 1949 and had to take a 2-year off from school. Because of his perseverance, he managed to get into Shanghai Kwang Hua Experimental High School (上海市私立光实中学) in 1951. After the China National College Entrance Examination in 1954, Tong was enrolled in Beijing Medical College. During his clinical internship in the emergency room in 1958, he was unfortunate to be infected with epidemic hepatitis, thus off from school for almost half a year and therefore lost the opportunity of becoming a physician. Instead, he tried hard to get admitted to Beijing Medical College graduate school, majoring in biochemistry. Tong focused on the metabolism and toxicity of blood ammonia in mice with liver tumors mentored by Professor Sizhi Liu (刘思职) (Fig. 2). Tong completed his graduate study in 1964, and became a junior faculty member in the Department of Biochemistry, Beijing Medical College. He was promoted to associate professor in 1985 and professor in 1988.

## Research in the United States

In August 1978, Tanjun Tong excelled in the national examination for scholars to visit the USA organized by the Minister of Education of the People’s Republic of China, and became one of the 52 scholars to the USA in December 1978, the first group of visiting scholars to the USA ever since the founding of P.R. China. After the 3 months of brief language training sessions at American University in Washington, D.C., Tanjun Tong went to study at Johns Hopkins University in Baltimore, Maryland mentored by biophysical chemist Paul O.P. Ts’o (曹安邦). Tanjun Tong was studying the effects of interferons on cell growth, during which process he acquired interdisciplinary knowledge of cell biology and biophysics. He was further advised by colleagues that the postdoctoral program at NIH (the National Institute of Health) was more suitable for his academic background, and he then applied to NIH. Half a year later, he received a fellowship from NIH and embarked on his postdoctoral research at NIH in Bethesda, Maryland. He was in the Division of Biochemistry and Biophysics of NIH, which was established by biochemist Darrell Liu (刘德勇). Tanjun Tong’s research areas at NIH include the biochemical characterization of edible bird’s nests and growth factors pertinent to tumor proliferation. In 1981, Tanjun Tong received the visiting fellow certificate from NIH and then turned to China.

In 1986, he visited the University of California, Davis in California, and New York University in New York City. He found there were research institutions on cellular senescence, aging, and gerontology, but their research depth and scope were far behind that of cancer research. This further inspired him to expand his subsequent research focus to senescence and aging. He and his wife Professor Zongyu Zhang gradually commenced aging research based on the old mice in their lab in the 1980s. In 1987 he participated in the 5-year reunion and seminar for CUSBEA program scholars.

Decades later, Tanjun Tong recalled, “The years of studying abroad in the 1980s are unforgettable. That experience overseas greatly broadened my horizons and exposed me to various advanced academic ideas, which had far-reaching significance for my future scientific research work.”(Zhen et al., 2021).

## Cancer research and protein biochemistry

Professor Tanjun Tong focused on the metabolism and toxicity of blood ammonia and urea synthesis in mice with liver tumors in the 1960s and demonstrated the higher blood ammonia and the declined ammonia tolerance in rats with liver tumors compared with normal mice. Arginine, ornithine, and citrulline were found to alleviate ammonia toxicity and increase ammonia tolerance in mice with liver tumors (Tong and Liu, 1965).

He introduced cutting-edge protein biochemistry advancements into China including the protein biosynthesis process and the intricate transcriptional and translational regulations in mammals (Huang and Tong, 1987; Tong, 1975, 1987; Tong and Zhang, 1978, 1979). He also introduced the chalones, which were widely discussed in the early 1970s, a class tissue-specific protein that inhibits cell mitosis and helps to curb tumor growth (Biochemistry Teaching and Research Group of Basic Department, 1976; Tong, 1976). He showed the serums of normal mice, the serum of cancerous mice, and tumor ascites in direct contact with cancer cells were all able to inhibit the growth of Ehrlich ascites cancer cells, among which tumor ascites had the strongest inhibitory capacity (Tong and Chen, 1978, 1979). The inhibitory activity of tumor ascites exhibited specificity towards cancer cells *in vitro*, sparing other normal cells (Zhou et al., 1980).

A series of studies by Tong et al. were performed to characterize the DNA-binding proteins in different cancer cells and elucidated that several novel DNA-binding proteins were possible cancer biomarkers in mice and humans (Chen et al., 1983; Gong and Tong, 1991a, 1991b; Gong et al., 1983, 1984; Liu et al., 1989, 1990; Tong et al., 1985; Tong et al., 1983a, 1983b, 1984a, 1984b; Zhai et al., 1990). DNA-binding proteins in mice erythrocytes with malaria were also studied (Yang et al., 1986, 1987). A DNA-binding protein from human serum with chymotrypsin inhibition activity was purified and characterized (Yu et al., 1987). The clinical test for DNA-binding protein 64DP from human serum was performed by the ELISA method, and 64DP could bind to chromatin in human liver cells (Yu et al., 1988, 1991).

The effects of different growth factors on cancers were studied. Several types of cancer cells had distinct sensitivities to EGF (epidermal growth factor), and the sensitivities correlated with their tumor malignancies. EGF could promote cell growth of human fibroblast cells but had little effect on human sarcoma cells, and distinct effects on different types of mouse ascites carcinoma cells. The inhibition effect for cancer cell growth and migration by interferons was tested. Interferons could antagonize the effect of EGF on human fibroblast cells *in vitro* (Tong, 1983a, 1983b, 1983c, 1983d; Wu and Tong, 1990; Xu and Tong, 1988; Xu et al., 1988). It was shown that EGF elevated the enzymatic activity of DNA topoisomerase, which was widely involved in DNA replication and cell proliferation (Li and Tong, 1994b; Wang et al., 1992; Zhou and Tong, 1991). The EGF receptor gene expression in both young cells and aging cells of human fibroblast were not obviously affected by EGF addition, while EGF receptor gene expression was found to be greatly upregulated in malignant transformed cells (Tang et al., 1991; Zheng and Tong, 1991). EGF could stimulate the activity of chromatin-associated protein kinase (Huang et al., 1989). EGF was demonstrated to possess the induction effect on neu/HER-2 oncogene expression in mouse embryo fibroblast cells (Zheng and Tong, 1992). EGF treatment on mouse embryo fibroblast indicated the EGF regulatory control for increased expressions of several transcription factors (Peng et al., 1993c). EGF was demonstrated to specifically bind to two types of EGF-binding sites in embryo mouse fibroblast cells (Peng et al., 1993a). EGF bound to the receptor on the cell surface entered the cell via endocytosis and subsequently translocated from the cell membrane to the nucleus (Peng and Tong, 1993). It was putatively found the methylation levels of genomic DNA and a proto-oncogene were down-regulated by the prolonged treatment of EGF (Peng et al., 1993b). It was suggested EGF could stimulate the kinase activity of a cell cycle regulation protein both in normal mouse embryo fibroblast cells and malignant transformed cells (Cai and Tong, 1994a). It was investigated EGF stimulation could advance the S phase and shorten the entire cell cycle, and EGF exerted a direct stimulating effect on transcription in the isolated cell nuclei (Cai and Tong, 1994b; Peng et al., 1994). EGF and FGF (fibroblast growth factor) were both shown to activate the transcription of the tumor suppressor Rb gene, but not of tumor suppressor p53 gene (Li et al., 1994a, 1994b). The transcription activity effects of EGF on different RNA polymerases were identified in cell nuclei *in vitro* (Li and Tong, 1995b). The epidermal pentapeptide isolated from mouse epidermal cells was able to inhibit DNA synthesis in both normal and transformed malignant fibroblasts (Liu and Tong, 1993, 1995, 1999). NGF (nerve growth factor) could increase the transcription activity in isolated nuclei from mouse fibroblasts *in vitro* (Li and Tong, 1995a). It was also indicated NGF could impede the proliferation of mouse embryonic fibroblast cells (Wang and Tong, 1992).

A few oncogenes and potential targets for cancers were under biochemical and clinical analyses. The proto-oncogene v-sis expression was identified in certain ascites tumor cells from mice (Tong et al., 1990). The neu/HER-2 oncogene abnormalities such as rearrangement and amplification were found in stomach cancer cases in China (Jin et al., 1993; Li and Tong, 1994a). Rearrangement and amplification of neu/HER-2 oncogene in transformed malignant mouse embryo fibroblasts were found, but no point mutation was detected (Li et al., 1995a). The complete deletion or partial deletion of the Rb tumor suppressor gene was studied in clinical stomach cancer cases in China, providing helpful insights for tumor early diagnosis and gene therapy (Huang et al., 1993; Peng et al., 1992). The membrane-bound glycoprotein antigen A33 was primarily found in intestinal epithelia, as a potential target for colon cancer. It was observed that gut-enriched Krüppel-like factor could bind to the A33 promoter and upregulate the transcription of A33 (Mao et al., 2003).

The isolation and biochemical characterization of the glycopeptides in an edible bird’s nest were conducted (Tong and Liu, 1984, 1985). The Na^+^/K^+^-ATPase activities on erythrocyte membrane gradually increased during their first year in mice but greatly decreased after one and half years. The effects of different traditional Chinese medicines on Na^+^/K^+^-ATPase activities were also tested (Chen et al., 1986, 1987; Tong et al., 1986). As for the phosphorylation studies, the transformed cells by radiation resembling an *in vitro* tumor exhibited a higher level of tyrosine phosphorylation, and a separation method for phosphotyrosine was developed (Xu and Tong, 1989; Xu et al., 1987).

**Figure 3.**
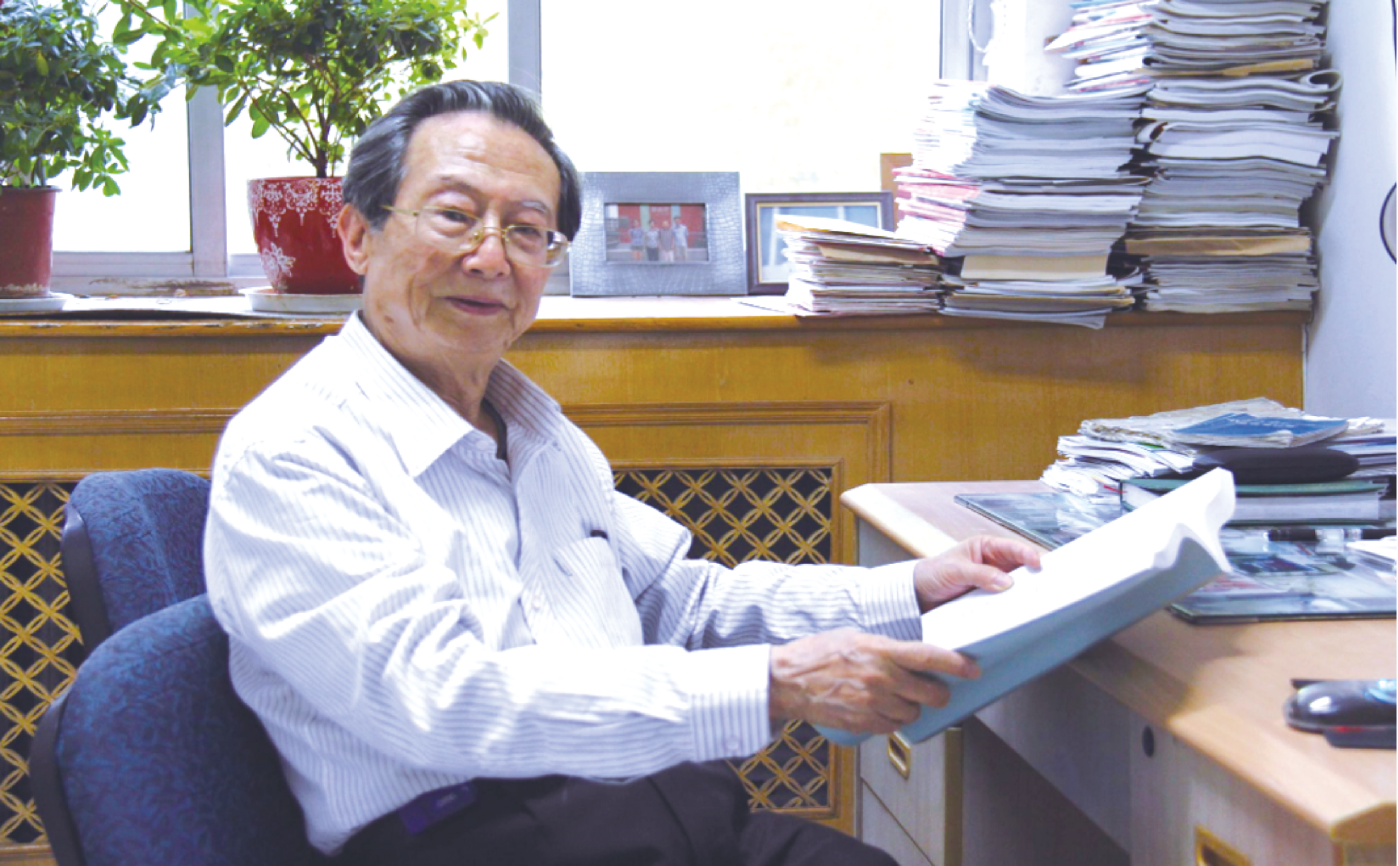
Professor Tanjun Tong at work.

Professor Tanjun Tong enthusiastically engaged in teaching the courses of biochemistry for undergraduates and advanced nucleic acid biochemistry for graduate students for over 50 years and still gave lessons to students even when he was 88 years old, respected by generations of students. He won the first prize for Outstanding Teaching Achievements of Universities in Beijing in 1993 (Fig. 3).

Due to the tremendous advances in biochemistry and molecular biology worldwide, Professor Tanjun Tong added a new chapter of protein biosynthesis to the Biochemistry textbook in the 1970s, exhibiting the latest progress in biochemistry of the previous two decades. This 1978 version of the Biochemistry textbook with novel and enriched contents fulfilled the urgent teaching requirement after the resumption of the China National College Entrance Examination. The textbook was revised several times in the following decades (Tong, 2003). He also participated in the compiling of another 30 textbooks and reference books in the fields of biochemistry, molecular biology, and gerontology.

## Explorations in cellular senescence and aging

Since the early 1990s, Professor Tanjun Tong has shifted his research focus to cellular senescence and aging, and extensively studied a plethora of research areas, encompassing telomere and telomerase, senescence regulating small molecules, senescence-regulator p16 and p21, senescence-related growth factors, mitochondria DNAs, and other senescence-associated genes and proteins.

Telomere and telomerase play a critical role in cellular senescence. The telomeres refer to the tandem repeat noncoding DNA sequences localized at the termini of linear chromosomes, and telomere shortening was recognized as the potential driver and hallmark of cellular senescence and aging. The telomerase is able to add telomere repeat nucleotide sequences onto the end of the chromosome, elongating the telomere. The discovery of telomerase was subsequently awarded the Nobel Prize in Physiology or Medicine in 2009. In contrast to the absence of telomerase activities in normal somatic cells, notable telomerase activities are observed in germ cells and most cancer cells. Maintaining the length of telomeres by telomerase was considered a mechanism for cancer cell proliferation. Professor Tanjun Tong observed the average telomere length of human fetal lung diploid fibroblasts was gradually shortened during sequential mitoses, with roughly 50 base pairs of telomere DNA loss per DNA replication round, indicating the telomere length could act as a biomarker for the human cell aging process (Zhang et al., 1997a). Similarly, it was discovered that the average telomere length of white blood cells from 180 human subjects was shortened by 35 base pairs per year and 1.38 kilobase loss in 40 years (Zhang et al., 1997b). Telomerase was a ribonucleoprotein complex, essentially composed of the reverse transcriptase protein components and the RNA template component. The reverse transcriptase protein components utilize the RNA template to synthesize tandem repeats of telomere DNA and add these repeats onto the chromosome termini. In MCF-7 human breast cancer cells, he and colleagues introduced stable expression of antisense oligonucleotide via virus vector to inhibit the RNA template of telomerase, blocking its synthesis of telomere repeat, leading to telomere-based growth arrest and apoptosis. Phenotypic changes in breast cancer cells were also observed, such as reduced cell growth, and decreased colony formation. It was shown the telomerase activity in the cancer cells was significantly inhibited compared with the control cell lines, and moreover, substantial shortening of the telomere length was detected in the cancer cells. The above results suggested the antisense inhibition of telomerase might act as a powerful therapeutic strategy for cancer treatment (Zhang and Tong, 2000, 2001; Zhang et al., 1999, 2001a, 2001b, 2003). Additionally, telomere shortening, irreversible cell cycle replication arrest, senescent cell morphology, and DNA damage accumulation were also observed in young human diploid fibroblasts when they were treated with low-dose hydrogen peroxide, mimicking the chronic pathological oxidative stress conditions (Duan et al., 2005).

Professor Tanjun Tong and colleagues extracted two small molecule isomers HDTIC-1 and HDTIC-2 from the effective traditional Chinese anti-aging herb Astragalus. Both isomers were capable of delaying the senescence of human fetal lung diploid fibroblasts. The isomers could prevent the cells from senescent morphology and push the cells in G_0_ or G_1_ phase into S phase for cell proliferation (Wang et al., 2003). p16, an important cyclin-dependent kinase inhibitor, could arrest the cell cycle, suppress cell growth, and promote cellular senescence and aging (Duan et al., 2004). It was demonstrated the expression of p16 was strongly inhibited by both HDTIC isomers in human cells (Wang et al., 2008). Furthermore, it was found HDTIC-1 and HDTIC-2 would delay senescence in cells by slowing down the telomere shortening rate by 56% and 42%, respectively (Wang et al., 2010). Moreover, a specific phosphatidylinositol 3 kinase inhibitor was identified to induce senescence in human diploid fibroblasts (Li et al., 2003).

p16 (p16^INK4a^, or CDKN2A) and p21 (p21^WAF1^), as tumor suppressors, were negative regulators of the cell cycle and were also implicated to promote replicative senescence in cells.

The introduction of the p16 gene into MCF-7 human breast cancer cells or human fibroblast cells via liposome or retroviral transfection caused notable features of cellular senescence, such as telomere shortening, suppression of growth rate, cell cycle arrest, and senescent cell morphology (Duan et al., 2000; Liu et al., 2001). The methylation level of p16 tended to decline with cell aging (Chen et al., 2001). Additionally, the transfection of p16 into human diploid fibroblasts reduced the cellular sensitivity of apoptosis induced by oxidative stress of hydrogen peroxide (Han et al., 2004). The inactivation of p16 in human diploid fibroblasts by antisense p16 expression via retroviral vector increased the cell proliferative life span and imposed a significant delay of cellular senescence features. This indicated the therapeutic potential of antisense p16 to prevent cell aging (Duan et al., 2001). They further characterized the regulatory elements on the upstream promoter region of p16 and identified a novel negative regulatory element (Wang et al., 2001). E47 protein was found to bind to the promotor region of p16 to upregulate p16 expression, and silencing of E47 could inhibit p16 expression and delay the onset of senescent phenotype. Additionally, Id1 functionally interacted with E47 to regulate p16 expression (Zheng et al., 2004). It was also discovered the transcription factor Sp1 could bind to the p16 promoter to induce the transcription activation of p16 during cell aging (Wu et al., 2007; Xue et al., 2004). It was demonstrated the transcription factor PPARγ could bind to the p16 promoter and enhance the expression of p16 to accelerate cellular senescence (Gan et al., 2008). It was shown that Lsh (lymphoid-specific helicase) interacted with p16 promoter to repress p16 expression via recruitment of histone deacetylases, delaying cell senescence (Zhou et al., 2009). It was indicated that exposure of cells to hydrogen peroxide induced the elevation of p16 expression with declined RNA-binding protein AUF1 level (Guo et al., 2010). The transcription factor B-MYB could bind to the promoter region of p16 and repress p16 transcription, delaying the cellular aging process (Huang et al., 2011). The tumor suppressor p33^ING1b^ could bind to the p16 promoter region to upregulate p16 expression, inducing cellular senescence (Li et al., 2011a). The transcription factor FOXA1 could significantly activate p16 transcription during cellular senescence, and FOXA1 antagonizes EZH2-mediated p16 repression in carcinogenesis (Li et al., 2013; Zhang and Tong, 2014).

During the process of sodium butyrate-induced apoptosis in human fibroblast, p21 expression was obviously down-regulated (Huang et al., 2004; Huang et al., 2000b). The introduction of exogenous p21 expression via retroviral vector transfection indicated signs of cellular senescence and reduced susceptibility to apoptosis induced by sodium butyrate (Huang et al., 2000a). The p21 promoter methylation suppressed p21 expression, postponing the aging process (Zheng et al., 2006).

Cellular senescence was suggested to be a progressive loss of the ability to respond to growth factor stimulation signals, and the underlying molecular mechanisms were also studied. Gradual aging of human diploid fibroblasts indicated a reduced responsiveness to either EGF or FGF stimulations (Li et al., 1995; Tang et al., 1994). As the fetal lung fibroblast cells aged, the induction susceptibility of proto-oncogenes c-fos/c-myc expression by EGF was reduced (Mao et al., 1997). The effects of EGF on a myriad of genes were assayed and widespread gene expression changes were observed, including the down-regulation of genes encoding membrane receptors and ion channels (Ma et al., 2002).

Mitochondria-related senescence studies were also performed. Mitochondrial DNA deletions were found to be associated with aging and more likely to occur in old mice, and cerebral ischemia further increased the occurrence of deletions (Zeng et al., 1999). In atherosclerosis-prone mice, mitochondrial DNA deletions were investigated to occur during the initiation stage of atherosclerosis, before plaque formation (Tian et al., 2016).

Multiple senescence-associated genes and proteins were also scrutinized, such as CSIG and Sirtuins.

Professor Tanjun Tong and colleagues identified the novel CSIG (cellular senescence-inhibited gene) protein, and CSIG significantly delayed the replicative senescence progression. CSIG inhibited tumor suppressor PTEN to promote cell proliferation through a translation suppression mechanism, and the ribosomal L1 domain and Lysine-rich region of CSIG were both functionally critical in this process (Ma et al., 2008, 2016). CSIG also promoted cell apoptosis in response to UV irradiation (Li et al., 2012; Zhao et al., 2012). It was suggested that CSIG could promote liver cancer cell proliferation by interaction and activation of the transcription factor c-MYC (Cheng et al., 2015). Large-scale screening of signaling pathway proteins modulated by CSIG was conducted using microarray analysis (Ma et al., 2015). In response to nucleolar stress, CSIG translocated to the nucleoplasm and interacted directly with MDM2 to be involved in the MDM2-p53 pathway (Xie et al., 2016). CSIG overexpression could repress NOLC1. NOLC1 expression was decreased in liver cancer tissues, while NOLC1 upregulation after CSIG knockdown promoted cell senescence and inhibited the proliferation of liver cancer cells (Yuan et al., 2017). It was unveiled that CSIG could delay cellular senescence and promote cell proliferation partially via PPARγ modulation (Jiang et al., 2022).

Sirtuins (SIRT 1–7), as a class of deacetylase, play a critical role in cell senescence regulation. Sirtuins were considered promising therapeutic targets for anti-aging diseases and were extensively studied by Professor Tanjun Tong. SIRT1 expression promotes cell proliferation and delays cellular senescence (Huang et al., 2008). It was shown that PPARγ inhibited SIRT1 at the transcriptional level (Han et al., 2010). Estradiol from female mice ovaries could upregulated SIRT1 by ubiquitination and degradation of PPARγ, partially explaining the higher life expectancy of females than males in mammals (Han et al., 2013). Calorie restriction could enhance SIRT1 protein stability by the reduction of ubiquitin-proteasome degradation of SIRT1, antagonizing the onset of senescence and age-associated diseases (Han et al., 2014). SIRT6 could decrease the acetylation level of tumor suppressor p27^KIP1^, leading to p27^KIP1^ ubiquitin-proteasome degradation, and delaying cellular senescence (Zhao et al., 2016). The RNA-binding protein hnRNP stabilized SIRT1 mRNA to increase SIRT1 expression, delaying senescence-associate secretory phenotype (Wang et al., 2016). The specific long noncoding RNA transcript, frequently upregulated in human liver cancer cells, could upregulate the expression of SIRT1 (Zhao et al., 2019).

Professor Tanjun Tong and colleagues identified TOM1 (target of Myb1) as a regulator of senescence progression in human lung diploid fibroblasts, which had not been previously associated with cellular senescence (Guo et al., 2004). They discovered α2M (alpha-2-macroglobulin) to be a reliable biomarker for cellular aging in cells and also characterized a novel positive transcription regulatory element within α2M promoter upregulating α2M during replicative senescence (Li et al., 2011b; Ma et al., 2004). They found the Leo1-like domain of the RDLp (replicative senescence down-regulated Leo1-like protein) accelerated the senescence of fibroblasts (Zhao et al., 2005). It was demonstrated that WWP1 (WW domain-containing E3 ubiquitin protein ligase 1) overexpression could delay cellular senescence by promoting tumor suppressor p27^KIP1^ proteasome degradation (Cao et al., 2011). Lsh was implicated in premature aging and cell senescence delay, and the E2F1 transcription factor could bind to Lsh promoter to activate Lsh protein expression (Niu et al., 2011). E2F1 transcription factor would bind to CSIG promoter and stimulate CSIG expression in growing cells, but the Rb protein could selectively mediate heterochromatin formation to disrupt the E2F1 binding site and block CSIG expression during cell senescence (Chen et al., 2012). Oncogenic Ras activation increased the intracellular reactive oxygen species (ROS), triggering replicative senescence. PKD1 (protein kinase D1) was identified as a downstream effector of ROS signaling to mediate senescence phenotype, and PDK1 deficiency promoted tumorigenesis (Wang et al., 2014). Overexpression of GTP-binding protein nucleostemin was found in most liver cancer tissues, which delayed cellular senescence, and the knockdown of nucleostemin promoted cell apoptosis in response to stress, indicating nucleostemin might be a potential therapeutic target for liver cancers (Yuan et al., 2015). TIFA (TRAF-interacting protein with forkhead-associated domain) protein was identified as a novel regulator for DNA damage-induced NF-κB activation, which was involved in carcinogenesis and cellular senescence (Fu et al., 2018).

**Figure 4.**
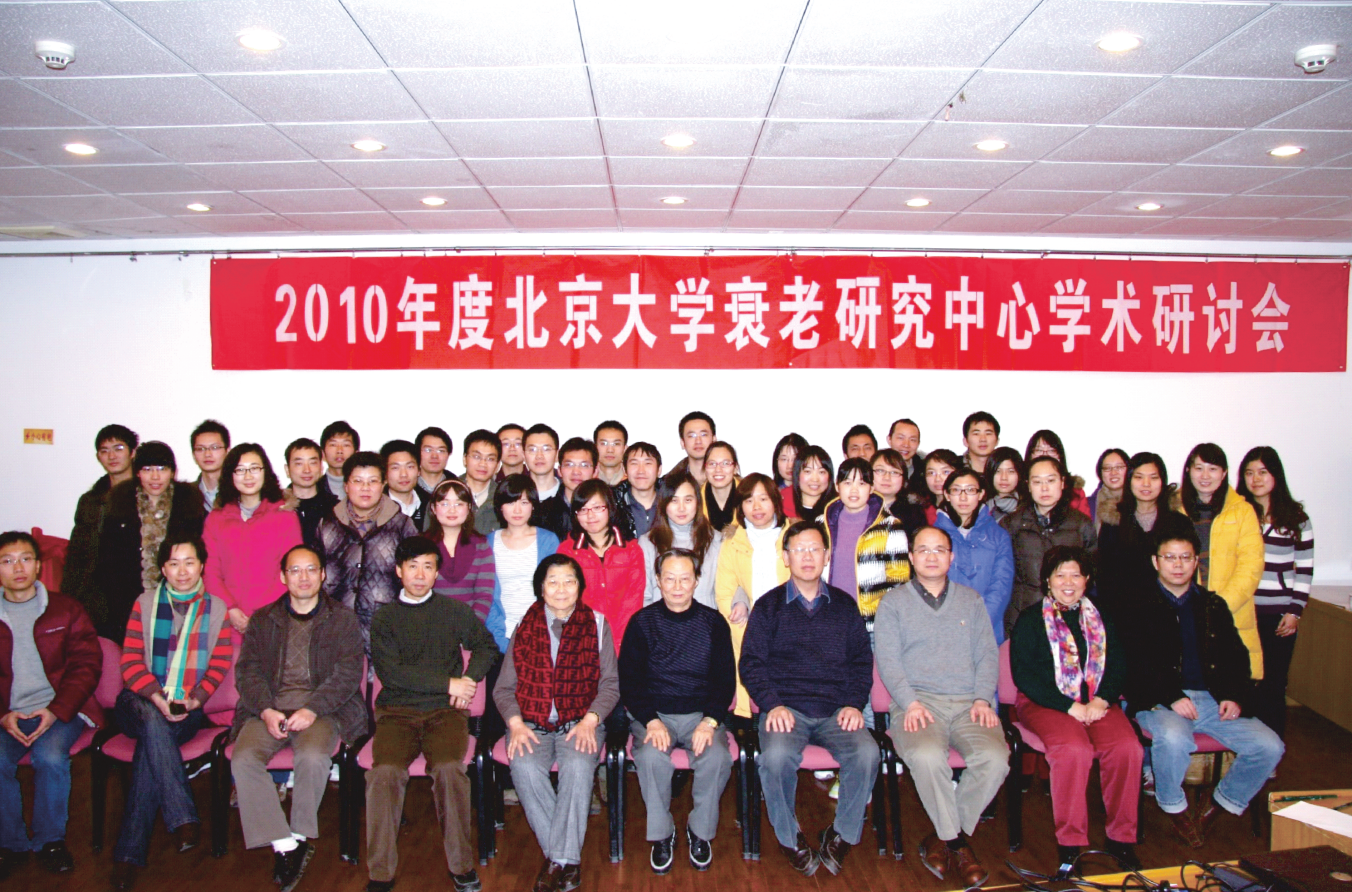
Academic seminar of Peking University Research Center on Aging in 2010, Professor Tanjun Tong (first row, left sixth), Professor Zongyu Zhang (first row, left fifth).

In 2004, Peking University Research Center on Aging headed by Professor Tanjun Tong was established, aiming at interdisciplinary studies among famous institutions, in cooperation with multiple hospitals (Fig. 4). He led a series of National Programs on Key Basic Research Projects (973 Programs), and other important research projects. His research on the molecular mechanism of cellular senescence was awarded the second prize of the Science and Technology Progress Award of the Ministry of Education of P.R. China in 1999, the second prize of the Chinese Medical Science and Technology Award in 2002, the first prize of Beijing Science and Technology Award in 2009, and the second prize of the China National Science and Technology Progress Award in 2010. Furthermore, his research on “mechanism of action and negative regulation of the dominant gene p16 in human cell senescence” ranked first in the Top Ten Breakthroughs of Science and Technology of Chinese Universities in 2002, and the research of “preliminary unveiling of the mystery of human cell aging” won the China Top Ten Breakthroughs of Science and Technology in 2002.

## Gerontology studies and social responsibilities

In response to the social challenges of population aging, Professor Tanjun Tong and his wife Professor Zongyu Zhang led the compilation of the book entitled *Medical Gerontology*: *Aging and Longevity* in 1995 (Tong and Zhang, 1995). The book primarily comprised the two parts of basic medicine and clinical medicine, exhibiting the research advances in medical gerontology, especially the significant role of molecular mechanism elucidation. This book was one of the first representative monographs that integrated the basic medical research of molecular biology and cell biology, nutritional advice, nursing and daily care, clinical diagnosis, and medication guidance for geriatric diseases. The book was updated several times and further incorporated the analyses of longevity genes, aging genes, and mitochondrial DNA damage by reactive oxygen species. They also published a book titled *Healthily Live One Hundred Years with Enjoyment*, which was intended for a general audience in 2006 (Zhang and Tong, 2006). To make the book's contents more readable and convincing, they interviewed 9 long-lived scholars, the youngest of whom was 81 years old and the oldest was 106 years old, sharing their secrets for longevity, including life routines, habits of nutritional diet and physical activities, and psychological personality traits. They also held lectures in the communities introducing the scientific knowledge of gerontology and aging.

Professor Tanjun Tong realized the urgent necessity to promote gerontology knowledge to the greater general public and established the website “China Elderly Healthcare Portal” with a team of young students in 2003. The website included both articles that were easy to comprehend by the public and professional academic discussions on aging and gerontology. The website received a great deal of attention from many aspects of society, and website visits quickly reached hundreds of thousands within just a few days.

Professor Tanjun Tong and his wife Professor Zongyu Zhang donated 500 thousand yuan to the School of Basic Medical Sciences at Peking University in 2013 for the Young Teachers Science and Technology Achievement Award.

Professor Tanjun Tong initiated the reform of China's postdoctoral system with enormous social responsibilities for science development in China. He commented, “The postdoctoral group is the vanguard and backbone of the science and technology front in various countries.”(Zhen et al., 2021). However, there were several shortcomings for China postdoctoral system at that time, such as the insufficiency for the number of postdoctoral sites and the number of postdoctoral researchers compared with the developed countries. He extensively reviewed a large volume of documents and meticulously analyzed the postdoctoral system in China, and then he drafted the reform plan for the China postdoctoral system along with several other academicians of the Chinese Academy of Sciences, proposing several suggestions for the reform. First, the competition and incentive mechanism could be implemented with the postdoctoral stipends determined by seniority and performance. Second, the two-year time limit for a term of the postdoc research could be more flexible, depending on the academic needs. Third, the number of postdoctoral researchers in China should increase and be flexible according to the requirement of principal investigators. The reform plan was entitled “Suggestions on reform of the postdoctoral system and expansion of the postdoctoral team,” and proposed to the State Council of the People’s Republic of China in 2009 (Tong et al., 2009). Then the postdoctoral system of China underwent substantial and effective changes in the next 10 years just as Professor Tanjun Tong had envisioned.

## The purpose of the cellular senescence studies by my team is to fundamentally understand the causes and mechanisms of human aging from the source

Professor Tanjun Tong relocated his research focus from cancer to aging, and pointed out that cancer cells with uncontrolled proliferation and aging cells with proliferative exhaustion both involved the molecular regulations of cells, “like the two sides of the same coin”. He said, “The purpose of the cellular senescence studies by my team is to fundamentally understand the causes and mechanisms of human aging from the source.” He devoted his life to seeking scientific excellence and pursuing medical benevolence, rather than fame or wealth. As his name indicated, Professor Tanjun Tong practiced the ideals of Confucianism all his life, “The exemplary gentleman is poised and unperturbed with magnanimity (君子坦荡荡).” He always said, “There’s more I want to do”, but he could no longer. We were profoundly saddened by his loss and his moral spirit will guide us for further academic pursuit of excellence.
